# The proteome of extracellular vesicles of the lung fluke *Paragonimus kellicotti* produced in vitro and in the lung cyst

**DOI:** 10.1038/s41598-023-39966-x

**Published:** 2023-08-22

**Authors:** Lucia S. Di Maggio, Kerstin Fischer, Devyn Yates, Kurt C. Curtis, Bruce A. Rosa, John Martin, Petra Erdmann-Gilmore, Robert S. W. Sprung, Makedonka Mitreva, R. Reid Townsend, Gary J. Weil, Peter U. Fischer

**Affiliations:** 1 Division of Infectious Diseases, Department of Medicine, Washington University School of Medicine, St. Louis, MO USA; 2 Department of Internal Medicine, Washington University of St. Louis School of Medicine, St. Louis, MO USA; 3 Division of Endocrinology, Metabolism and Lipid Research, Department of Medicine, Washington University School of Medicine, St. Louis, MO USA; 4 Department of Cell Biology and Physiology, Washington University School of Medicine, St. Louis, MO USA

**Keywords:** Proteomics, Parasitology

## Abstract

Paragonimiasis is a zoonotic, food-borne trematode infection that affects 21 million people globally. Trematodes interact with their hosts via extracellular vesicles (EV) that carry protein and RNA cargo. We analyzed EV in excretory-secretory products (ESP) released by *Paragonimus kellicotti* adult worms cultured in vitro (EV ESP) and EV isolated from lung cyst fluid (EV CFP) recovered from infected gerbils. The majority of EV were approximately 30–50 nm in diameter. We identified 548 *P. kellicotti*-derived proteins in EV ESP by mass spectrometry and 8 proteins in EV CFP of which 7 were also present in EV ESP. No parasite-derived proteins were reliably detected in EV isolated from plasma samples. A cysteine protease (MK050848, CP-6) was the most abundant protein found in EV CFP in all technical and biological replicates. Immunolocalization of CP-6 showed strong labeling in the tegument of *P. kellicotti* and in the adjacent cyst and lung tissue that contained worm eggs. It is likely that CP-6 present in EV is involved in parasite-host interactions. These results provide new insights into interactions between *Paragonimus* and their mammalian hosts, and they provide potential clues for development of novel diagnostic tools and treatments.

## Introduction

*Paragonimus* lung flukes cause paragonimiasis, a food-borne neglected tropical disease (NTD) that affects more than 20 million people in Asia, Africa, and the Americas. Paragonimiasis is a significant public health problem in China and south-east Asia where it is referred to as “oriental lung fluke infection”^1^. Paragonimiasis is a zoonosis and common in animals that eat freshwater crustaceans. About 50 species of the genus *Paragonimus* have been described, and about one third of them have been shown to cause disease in humans. Symptoms of paragonimiasis may include chest pain, abdominal pain, cough, fever, weight loss, and hemoptysis. Paragonimiasis patients often have abnormal chest x-ray findings with pulmonary nodules, focal infiltrates, and pleural effusions. These clinical and x-ray findings explain why paragonimiais is sometimes confused with pulmonary tuberculosis or lung cancer^2^. Infections are most common in areas where people eat raw or poorly cooked freshwater crustaceans that contain infective parasite larvae called metacercariae that penetrate the gut and migrate from the peritoneum through the diaphragm and pleural space into the lung. While the infection can be treated with praziquantel, diagnosis of paragonimiasis is often delayed because clinical diagnosis is not specific, detection of fluke eggs in sputum or stool is insensitive, antibody assays do not differentiate between past and present infection, and antigen tests are not available.

Excretory/secretory products (ESP) released by tissue helminths can interact with the host immune system and tissues, and they may contain biomarkers for diagnosis. Our studies have focused on *Paragonimus kellicotti,* the agent of North American paragonimiasis, because metacercariae can be easily collected in the USA, they develop into adult worms in gerbils, and because extensive genome and transcriptome data are available for this species^3–5^. In a recent study, we used mass spectrometry to study the proteome of adult stage *P. kellicotti,* their ESP when cultured in vitro, and ESP found in the lung cyst of infected gerbils (cyst fluid proteins, CFP)^6^. This was a significant step toward identification of potential biomarkers for paragonimiasis, but more work was needed. For example, ES products released in in vitro culture may not resemble the proteins produced in vivo, culture supernatants may be contaminated with proteins originating from dead or disintegrating flukes, and sensitivity for detection of *P. kellicotti* proteins in cyst fluid is hampered by a huge abundance of host proteins.

Helminths produce extracellular vesicles (EV) that contain nucleic acid and proteins^7^. Isolation of EV allows analysis of the parasite products without significant contamination with host proteins^8^. EV are 40–1000 nm membrane-bound delivery pods released from cells that have been implicated in many biological processes including the mediation of intercellular communication and immune responses^9–11^. Currently, EV are subdivided into 2 major groups of interest, namely microvesicles (MV) and exosomes, which are distinguished from one another by size, site of biogenesis and content.

EV produced by *Paragonimus* parasites have not previously been subjected to proteomic analysis^7^. However, EV from other food-borne trematode species have been studied. For example, EV of the liver fluke *Fasciola hepatica* contain proteins that modulate host dendritic cell phenotype and activity^12^. Surface molecules on *F. hepatica* EV direct their uptake by post cells^13^, and the EV contain proteins involved in pathogenesis^14^. The south-east Asian liver fluke *Opisthorchis viverrini* secretes EV that promote carcinogenesis^15^.

The aim of the present study was to assess the protein content of *P. kellicotti* EV released by adult flukes in vitro or in vivo in lung cysts. We established protocols to isolate EV in different sample types and visualized parasite EV by electron microscopy. We tested whether proteins present in EV have value as diagnostic biomarkers. We identified a cysteine protease (CP-6) as a major antigen of EV and localized this protein not only in the tegument of the adult parasites but also in the lung cyst and adjacent tissues in experimentally infected gerbils.

## Results

### *P. kellicotti* release EV as part of total ESP

**Figure 1:**
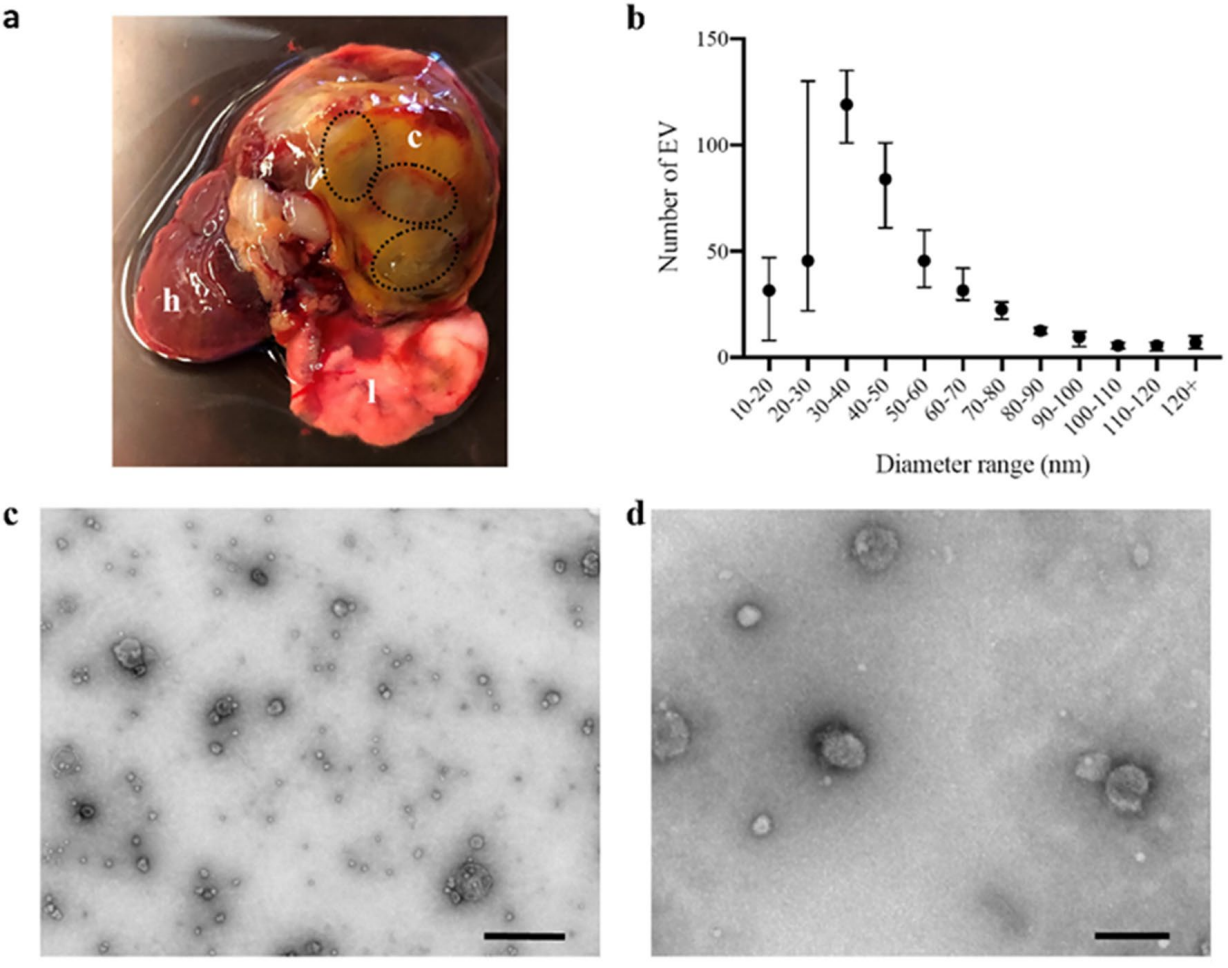
Size characterization of EV detected in *P. kellicotti* ESP. *P. kellicotti* were cultured for 24–72 h at 37 °C in 5% CO2. EV were then purified by SEC from the ESP. The profile of these isolated EV was assessed using a 1200 EX II TEM and ImageJ software. The majority of the EV in the sample fall within the size range of exosomes. (**a**) Heart and lungs of a gerbil infected with *P. kellicotti*. Three flukes (dotted circles) were recovered from one cysts (c) that occupies an entire lobe of the lung. Heart (h), healthy lung (l). (**b**) Four different images were analyzed to estimate EV size; the median is represented in the graphic (black point) and bars represent the range. (**c**) TEM 10,000× magnification. Scale bar represents 500 nm. (**d**) TEM 40,000× magnification. Scale bar represents 100 nm.

*P. kellicotti* parasites were cultured in vitro for 24–72 h in order to allow EV release. EV were isolated and purified from the ESP by size exclusion chromatography (SEC). The size of these EV preparations was assessed by electron microscopy. The average EV size was calculated from four separate captures (Fig. 1b–d). The majority of the EV in the sample were approximately 30–50 nm in size. However, the analysis also noted the presence of a small population of EV with a size range of 10–20 nm (Fig. 1b) and another at 90–120 nm. Particles that are 100 nm or smaller fall in the size range of exosomes^16^. Thus, membrane-enclosed entities with sizes and morphological features consistent with EV were observed by EM in all *P. kellicotti* ESP EV samples.

### Proteomic characterization EV released from *P. kellicotti*

**Figure 2:**
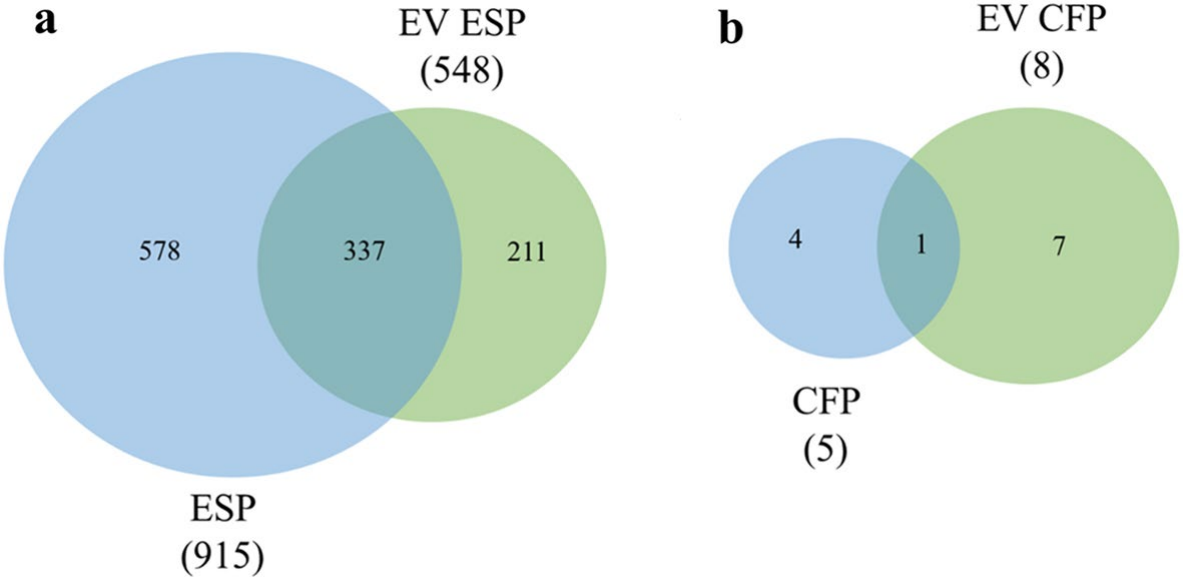
Comparison between samples. (**a**) Number of proteins for ESP (Di Maggio et al. 2022) versus EV ESP (present study). (**b**) Number of proteins for CFP versus EV CFP. *CFP* cyst fluid proteins, *ESP* excretion/secretion products, *EV* extra-vesicles.

The total number of proteins found for the three samples types is summarized in Table 1. These results were trimmed to only include proteins that appeared in two replicates and were supported by at least two different peptides (Table 1 and Supplementary Table S1). This analysis identified 548 *P. kellicotti*-derived proteins in EV ESP (Fig. 2 and Supplementary Table S2). The identified proteins included several proteases, oxidases, reductases, heat shock proteins and cytoskeletal associated proteins. Table 3 shows the functional enrichment analysis of 145 proteins (identified with more than 10 unique peptides) that were detected in EV ESP. The enrichment analysis identified the classes of proteins that were over-represented in the EV ESP. The three most highly represented GO terms in the molecular function category were ATP hydrolysis activity, ATP-dependent activity and ribonucleoside triphosphate phosphatase activity (p = 7.7 × 10^–8^, p = 2.3 × 10^–7^ and p = 2.8 × 10^–7^, respectively). The three most significant overrepresented InterPro domains were chaperones (p < 10^–5^) and GroEL-domains (p = 1.1 × 10^–4^). Among the KEGG domains, the most represented were exosome proteins, chaperones and membrane trafficking domains (p < 10–6) (Table 3). These proteins belong to processes related to catalytic activity, cytoskeleton associated proteins and signal transduction. We only found eight proteins in the EV CFP; seven of these were also detected in EV ESP (cysteine protease-6, yolk ferritin, one unknown protein, clathrin, ATP-binding cassette subfamily D, V-type proton ATPase and transitional endoplasmic reticulum ATPase, see Table 2, Supplementary Table S1). The only protein detected in EV CFP but not in EV ESP was a lysosomal pro-X carboxypeptidase (Table 2). Among the proteins present in EV CFP, CP-6 was the most commonly represented protein in all the technical and biological replicates for this sample type (Table 2). From the total number of proteins identified only 80 have a predicted signal peptide, three being found in EV CFP and 27 in EV ESP sample (Supplementary Table S1). EV ESP and EV CFP samples share 2 of these proteins (CP-6 and ABC transporter-like with accession numbers AZZ10060.1 and KAF6775698.1, respectively). No parasite-derived proteins that met our detection requirements were found in the GBP (Supplementary Table S1; Table 3).

**Table 1:** Total number of peptides and proteins found in all EV samples.

Sample type	Isolation method	Total numbers after removing host peptides		Total numbers with 2 peptides and in 2 replicates	
		Peptides	Proteins	Peptides	Proteins
GBP	ME kit	18	12	0	0
CFP	SEC	220	82	40	8
ESP	SEC	10,750	1270	4233	541

Numbers after removing the peptides that match with the host.

*GBP* gerbil blood proteins, *CFP* cyst fluid proteins, *ESP* excretion/secretion products, *SEC* size exclusion chromatography, *ME kit* exosome isolation kit.

**Table 2:** Proteins found in the EV of CFP.

Accession number	Annotation	Unique peptide across samples				Unique spectra count across samples				Similarity % (specie, accession number)
		CFS 1	CFS 2	CFS 3	CFS 4	CFS 1	CFS 2	CFS 3	CFS 4	
AZZ10060.1	cysteine protease-6 partial	4	12	6	12	4	78	15	58	89.47% *Paragonimus pseudoheterotremus* (AOH96646.1)
AZZ10063.1	yolk ferritin partial	1	4	3	1	1	4	3	2	93.4% (*Paragonimus heterotremus*, KAF5394626.1 )
KAF6779441.1	Clathrin heavy chain	1	2	0	2	1	14	0	4	97.76% (*Paragonimus westermani,* KAF8567126.1)
KAF6771971.1	Unknown protein	1	3	3	0	1	3	4	0	87.39% (*Paragonimus skrjabini miyazakii*, KAF7259151.1)
KAF6775556.1	Lysosomal Pro-X carboxypeptidase	0	1	2	2	0	1	2	2	94.03% (*Paragonimus skrjabini miyazakii*, KAF7255109.1)
KAF6776640.1	Transitional endoplasmic reticulum ATPase partial	0	2	2	0	0	8	4	0	99.07% (*Paragonimus skrjabini miyazakii*, KAF7256594.1)
KAF6775698.1	ATP-binding cassette subfamily D (ALD) member 4	2	2	0	0	2	4	0	0	82.91% (*Paragonimus westermani,* KAA3673521.1)
KAF6776298.1	V-type proton ATPase catalytic subunit A	2	2	0	0	2	2	0	0	98.50% (*Paragonimus westermani,* KAF8569881.1)

Accession numbers of each of the 8 parasite-derived proteins found in the EV CFP sample are in the first row followed by the annotation. The GenBank accession number of the top blast hit and it similarity to the match is given in parenthesis. The proteins are ordered from the most to the least abundant in the EV CFP replicates.

*CFP* cyst fluid proteins.

**Table 3:** Significantly enriched gene ontology molecular function pathways, Interpro and KEGG domains.

Gene ontology molecular function		Number of proteins in *P. kellicotti* database	Number of detected proteins in the EV ESP	FDR-adjusted P value
GO:0016887	ATP hydrolysis activity	85	14	7.7 × 10^–8^
GO:0140657	ATP-dependent activity	309	24	2.3 × 10^–7^
GO:0017111	ribonucleoside triphosphate phosphatase activity	199	19	2.8 × 10^–7^
GO:0016462	pyrophosphatase activity	210	19	5.2 × 10^–7^
GO:0016818	hydrolase activity, acting on acid anhydrides, in phosphorus-containing anhydrides	217	19	6.3 × 10^–7^
GO:0016817	hydrolase activity, acting on acid anhydrides	218	19	6.3 × 10^–7^
GO:0051082	unfolded protein binding	28	7	1.2 × 10^–5^
GO:0035639	purine ribonucleoside triphosphate binding	798	34	7.1 × 10^–5^
GO:0032555	purine ribonucleotide binding	801	34	7.1 × 10^–5^
GO:0032553	ribonucleotide binding	804	34	7.1 × 10^–5^
*KEGG domains*				
4147	Exosome	533	53	0
4131	Membrane trafficking	806	44	0
3110	Chaperones and folding catalysts	165	16	9.1 × 10^–6^
4144	Endocytosis	163	13	1.1 × 10^–3^
4612	Antigen processing and presentation	46	7	1.8 × 10^–3^
710	Carbon fixation in photosynthetic organisms	28	5	1.0 × 10^–2^
4141	Protein processing in endoplasmic reticulum	134	10	1.2 × 10^–2^
5134	Legionellosis	33	5	1.7 × 10^–2^
*Interpro domains*				
IPR002194	"Chaperonin TCP-1, conserved site"	6	5	1.7 × 10^–5^
IPR017998	Chaperone tailless complex polypeptide 1 (TCP-1)	8	5	7.7 × 10^–5^
IPR027413	GroEL-like equatorial domain superfamily	9	5	1.1 × 10^–4^
IPR027410	TCP-1-like chaperonin intermediate domain superfamily	10	5	1.7 × 10^–4^
IPR002423	Chaperonin Cpn60/GroEL/TCP-1 family	11	5	2.0 × 10^–4^
IPR027409	GroEL-like apical domain superfamily	11	5	2.0 × 10^–4^
IPR000308	14-3-3 protein	9	4	2.8 × 10^–3^
IPR023410	14-3-3 domain	10	4	3.4 × 10^–3^
IPR013126	Heat shock protein 70 family	33	6	3.4 × 10^–3^
IPR001464	Annexin	4	3	3.4 × 10^–3^

Top 145 *P. kellicotti*-derived proteins detected in the EV ESP sample were used, these are the proteins that were supported by 10 or more unique peptides. The top 10 enriched terms for each functional category are shown.

Among the identified proteins, the most abundant proteins in the EV ESP, based on spectra count, were myoferlin, calcium-binding protein, lysosomal alpha mannosidase, charged multivesicular body protein 2a and major vault protein. Proteins involved in vesicle trafficking (annexin, tetraspanin, myoferlin, charged multivesicular body protein 2a, Rab protein family, and vacuolar protein sorting-associated protein among others) were found in the EV ESP (Supplementary Table S1).

### Comparison of EV proteomics results with results from total CFP and ESP samples

We compared the current EV proteomic results with our previous CFP and ESP results obtained with the same methodology^6^. From the 915 *P. kellicotti*-derived proteins found in the ESP in a previous study (Supplementary Table S2), 337 were found in the EV ESP that is 60.8% of the proteins present in the EV ESP (Fig. 2a). Most of these were among the most abundant proteins in both samples (e.g., heat-shock proteins, cytoskeletal proteins and some uncharacterized proteins. see Supplementary Table S2). Total CFP contained 5 *P. kellicotti*-derived proteins that met our requirement of at least 2 peptides in at least 2 replicates and only one of these proteins (cysteine protease-6) was also detected in EV CSP: (Fig. 2b and Supplementary Table S3). This CFP was analyzed with a database that did not contain the CP-6 protein sequence (accession number AZZ10060.1). However, that database contained two proteases (accession number KAF6777412.1 and KAF6769383.1) that are similar to AZZ10060.1 (Supplementary Fig. S1). Apart from that, the peptides founded for both sequences KAF6777412.1 and KAF6769383.1 match with the sequence for AZZ10060.1. KAF6777412.1 has a repeat sequence of 53 amino acids (Supplementary Fig. S2, in blue) that appears again at the end of the sequence.

### Localization of CP-6 in the parasite and in infected lung tissue

**Figure 3:**
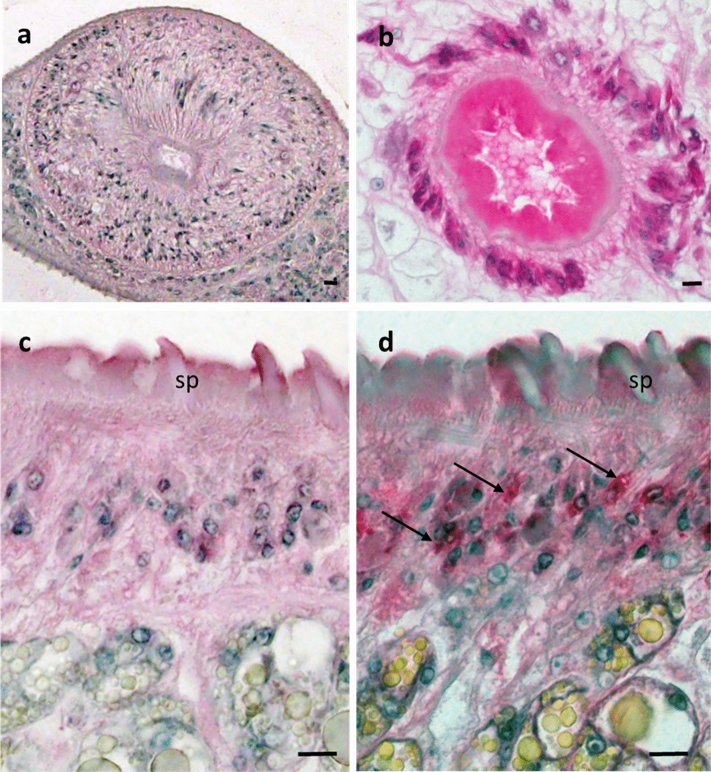
Immunolocalization of CP-6 in the lung fluke *P.kellicotti.* Oral sucker (**a**) and tegument (**c**) stained with preimmune serum served as negative controls. Intense red staining for CP-6 was found in the sucker (**b**) and the cellular layer (arrows) below the spines (sp) of the tegument (**d**). Scale bar represents 20 µm.

**Figure 4:**
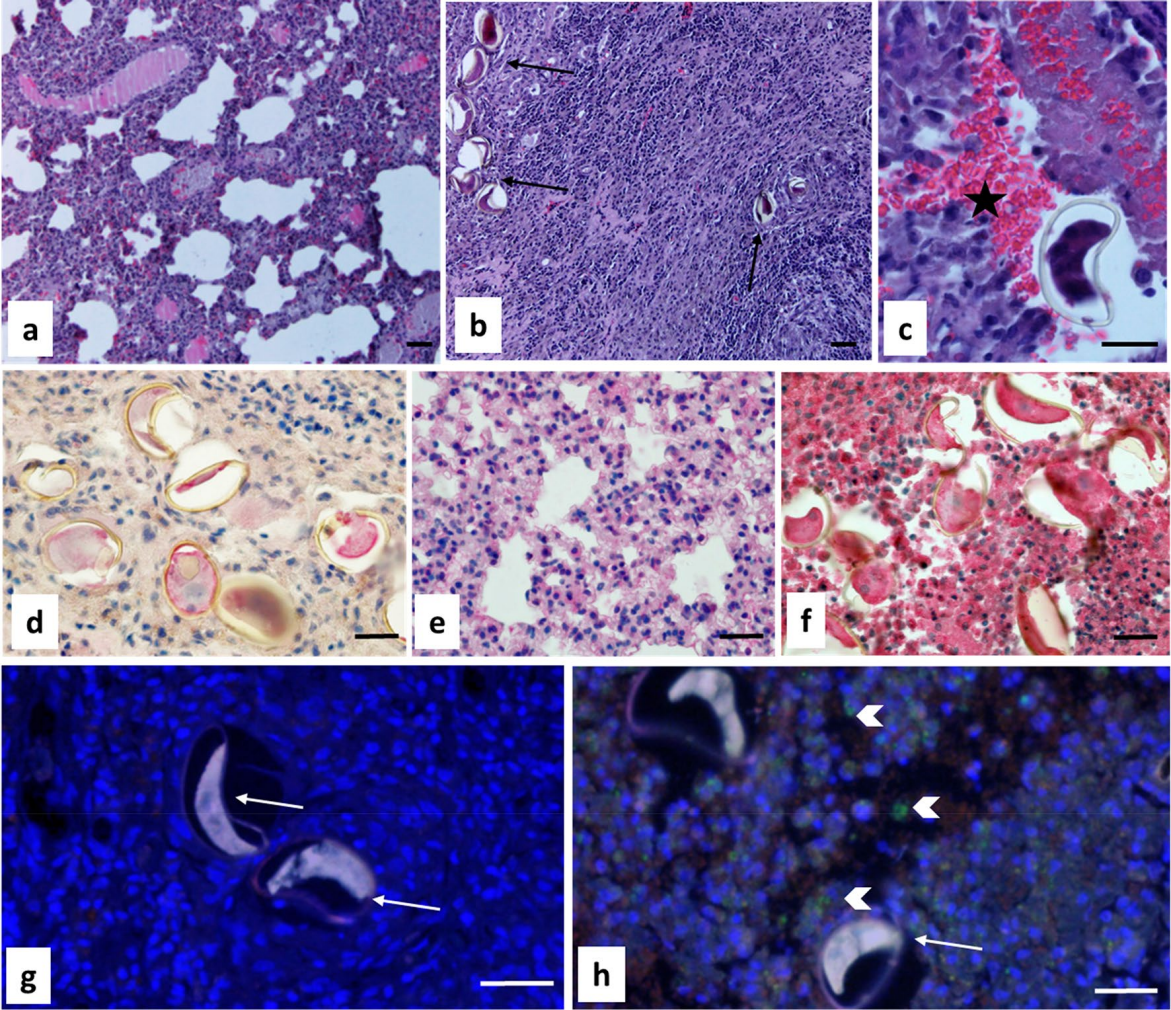
Immunolocalization of CP-6 in lung cyst tissue of a gerbil infected with *P. kellicotti*. (**a**–**c**) H&E stain showing lung tissue of an uninfected gerbil (**a**), lung cyst tissue of an infected gerbil with multiple *P. kellicotti* eggs (**b**, arrows). (**c**) Shows an egg at higher magnification next to a collection of red blood cells (asterisk). (**d**) Shows parasite eggs and lung cyst tissue labeled with preimmune mouse serum. (**e**) Shows intact lung tissue labeled with mouse anti-CP-6 antibody. (**f**) Shows parasite eggs and lung cyst tissue stained for CP-6. Intense labeling (red) can be seen in both in the cyst tissue and the eggs. (**d**–**f**) use mouse anti-human as secondary antibody and Magenta (Dako) as chromogen. (**g**,**h**) show immunofluorescence labeling of lung cyst tissue and parasite eggs (arrows). (**g**) was labeled with preimmune serum. (**h**) was labeled with mouse anti-CP-6 antibody shows green fluorescence (arrowheads). Scale bar represents 20 µm.

No signal was observed in worm sections when pre-immune serum was used as the primary antibody (Fig. 3a, c). In contrast, specific staining for CP-6 was observed in the oral sucker (Fig. 3b) and the cellular layer of the tegument below the outer layer with the tegumental spines (Fig. 3d). Routine histology with hematoxylin and eosin showed that the tissue of uninfected gerbils and the healthy looking lungs without cysts of infected gerbils looked quite different from the lung cyst tissue from infected gerbils (Fig. 4a–c). Intense blue staining for nuclei was observed in the tissue of lung cysts that contained the flukes and parasite eggs. Lung tissue was mostly replaced by a granulomatous infiltrate and no alveoli were visible in this area. A large number of operculated *P. kellicotti* eggs was observed in the lung cyst, scattered as individual eggs or small clusters of eggs. No specific tissue labeling was seen when the preimmune serum was used as primary antibody for control (Fig. 4d) or when the CP-6 antibody was used on lung healthy tissue (Fig. 4e). The most intense staining for CP-6 was seen in the infiltrated lung cyst tissue in the vicinity of *P. kellicotti* eggs (Fig. 4f). We also localized CP-6 in lung cyst tissue by immunofluorescence. No green fluorescence was visible after tissue was labeled with preimmune serum (Fig. 4g), but antibody to CP-6 resulted in a clear green signal (Fig. 4h).

## Discussion

We have analyzed the proteomes of EV released by adult *P. kellicotti* during in vitro culture and EV released in vivo in lung cysts of infected gerbils. For this purpose, we used SEC to separate exosomes and other EV from contaminating soluble proteins. The purity and size of the EV isolated from ESP was assessed by TEM; morphometrics detected mostly intact circular structures in the size range from 10 to 120 nm, with a peak at 30–40 nm. These findings indicated successful isolation of EV with a size and morphology that were consistent with EV isolated with similar methods from other helminth parasites such as *F. hepatica* (37–153 nm)^17^, *Brugia malayi* (85–200 nm)^18^ and *Schistosoma mansoni* (50–130 nm)^19^. SEC appears to be a safe and reliable method for efficient purification of EV produced by *Paragonimus* parasites.

Proteomic analysis of EV purified from *P. kellicotti* ESP detected proteins involved in cellular trafficking, heat-shock proteins, cytoskeletal associated proteins, 14-3-3, proteases, annexin and GAPDH. The Vesiclepedia exosome database shows that similar types of proteins have been identified in EV isolated from other organisms^20^. The presence of orthologous proteins in EV released from a variety of helminth species supports the validity of results from this study^17,19,21^ (Supplementary Figure S1). Several proteins found in the EV of ESP play key role in the survival of the parasite in the host. In the gerbil model for *P. kellicotti*, the metacercariae hatch in the intestine and penetrate the intestinal wall. Immature flukes migrate from the peritoneal cavity to the pleural space through the diaphragm and traverse the visceral pleura to invade the lung. This migration requires 3 to 4 weeks. In the lung, flukes induce the formation of cysts at the expense of functional lung tissue (Fig. 1a)^3^. The parasites must cross tissue planes, eat, and evade the host immune system as they migrant and establish residence in the lung and the proteins released in the EV can help with these functions^22^.

Our analysis of the ESP and CFP proteomes of *P. kellicotti* showed that approximately 90% of the proteins identified had no predicted signal peptide^6^. Extracellular proteins lacking a predicted signal peptide have also been commonly observed in ESP proteomes of a variety of helminth and protozoan parasite species^23–25^. These proteins are assumed to be excreted by a non-classical pathway such as secretion or excretion by the intestine, excretory pores, surface cuticle/tegument shedding or release of EV. In our study we found that 61.5% of the proteins in the EV ESP were also detected in the ESP (Fig. 2) and among them only 17 proteins have a predicted signal peptide (Supplementary Table S2)^6^. Proteins destined to enter the classical secretory system contain a signal peptide for translocation into the endoplasmic reticulum. However, proteins that lack signal peptides are actively packaged for secretion in alternative pathways as inside EV^26^.

The lack of suitable diagnostics for many parasites has led to an increased interest to identify parasite-derived molecules that are suitable biomarkers for infection^27^. Parasites release biologically active molecules into their hosts and some of these parasite-derived proteins are regularly found in the host’ plasma and sera during infection^8,28,29^. The role of exosome-derived proteins as biomarkers for helminth infection has been only gradually recognized^30^. Data on the EV produced by helminths in vivo are scarce. In the present study, we reliably detected eight parasite proteins in EV of CFP. Among them we found a transitional endoplasmic reticulum ATPase (Supplementary Table S1). This protein is involved in endoplasmic reticulum membrane homeostasis and ubiquitination, and it has been detected in EV released from cancer cells^31–33^. Moreover, this ATPase was one of the three parasite-derived proteins found in serum EV from *Echinococcus multilocularis*-infected mice at different times after infection^34^. The authors describe this protein as a biomarker candidate for early diagnosis of echinococcosis.

We detected the protease CP-6 (accession number AZZ10060.1)^6^ in EV CFP, total CFP, EV ESP and total ESP samples. This protein was also the most abundant protein in the EV CFP in all the replicates (Supplementary Table S1). In 2021, Curtis et al. evaluated the potential of recombinant CP-6 as an antigen for serodiagnosis of human paragonimiasis. An assay for IgG4 antibodies to CP-6 showed high sensitivity and specificity, and it detected antibodies in patients with early or mature infections with several *Paragonimus* species^35^. Our current study showed that this protein is localized in the tegument, vitelline follicles, oral sucker, spines and gut of *P. kellicotti* (Fig. 3b, d), and the protein was also present in host tissues near the parasite and eggs (cyst and adjacent lung). Additional studies will be needed to further understand the function of CP-6 in *Paragonimus* biology and to explore its potential as a drug target.

The second most abundant protein in the EV CFP was an egg yolk ferritin (accession number AZZ10063, Table 2). This protein was found in the ESP and EV ESP but not in the CFP (Supplementary Tables S1, S2 and S3). Ferritins are intracellular proteins involved in iron metabolism and have been described in several helminths parasites^36^. It has not been demonstrated that *Paragonimus* feed directly on blood but flukes can take up blood cells from the host^37^ and ferritin function can be related to feeding inside the cyst. A recombinant yolk ferritin from *P. westermani* has been studied as an antigen for antibody detection in subjects with cerebral paragonimiasis^38^. This ferritin shares 77% similarity with the *P. kellicotti* ferritin found in the EV CFP.

Structural proteins such as universal stress proteins (USP) were also detected in the EV ESP sample. This family of proteins has been described in helminths before. They help parasites to overcome unfavorable environmental conditions such as oxidative stress, temperatures and pH fluctuations^12,39^. In *Schistosoma* USP play a role in parasite survival especially during the egg hatching processes and for modulating musculature and motor activity, especially after exposure to praziquantel^40^. USP genes are not found in humans or other definitive hosts of parasitic helminths. Thus they could be useful for diagnosis and could represent therapeutic targets.

A limitation of the study is that many more parasite proteins are likely to be released in EV in vivo than the few we detected. Many are probably washed away or destroyed by the host, and others may not have been detected because of the excess of host proteins present even after enrichment of EV CFP by SEC.

We did not detect any parasite-derived proteins in EV GBP that met our requirements of 2 peptides in two different replicates (Supplementary Table S1). There are > 10,000 different proteins in mammalian plasma with a relatively small number of highly abundant plasma proteins^41^. Host EV concentrations are also high in plasma, and this makes it difficult to identify low concentrations of parasite proteins in EV enriched from plasma. More work will be required to overcome this challenge. To overcome this, several strategies might be used such as fractionation of digests (deep scale proteomics), enrichment, or labeling to make low-abundance proteins and peptides more visible^42,43^.

## Concluding remarks

Our study has shown that *Paragonimus* lung flukes produce EV that can be analyzed by mass spectrometry after enrichment. Many parasite proteins were identified in EV released by parasites cultured in vitro, and a smaller number of proteins were identified in EV released in vivo by flukes in lung cysts. The protease CP-6 was an especially abundant protein in both EV ESP and EV CFP, and it was also present in host tissues adjacent to flukes in the lung. We are convinced that improved understanding of host-parasite interactions through research on EV may lead to advances in the diagnosis and treatment of helminthic diseases.

## Materials and methods

### Ethics statement

Mongolian gerbils (*Meriones unguiculatus*) aged 5 to 8 weeks old were purchased from Charles River Laboratories (Worcester, MA, USA). They were kept under specific pathogen free conditions in an animal care facility of the Department of Comparative Medicine at Washington University in St. Louis. (Missouri, USA). All procedures involving animals were performed by specially trained personnel. Gerbils were euthanized by CO_2_ inhalation. Ethical permission for the use of animals in research was approved by Washington University’s Institutional Animal Care and Use Committee (IACUC) (protocol ID 20-0503). All methods described below were performed in accordance with the relevant guidelines and regulations. All methods are reported in accordance with ARRIVE guidelines (https://arriveguidelines.org).

### Preparation of *P. kellicotti* EV

*P. kellicotti* metacercariae were isolated from the heart tissue of two naturally infected species of crayfish (*Orconectes luteus* and *Orconectes punctimanus*) that were collected in Huzzah Creek (Missouri, USA) as previously described^3^. A total of four or five metacercariae, in 150µL phosphate buffered saline (PBS), were inoculated into each gerbil by intraperitoneal injection. 5 weeks post-infection infected animals were euthanized, and lungs were examined for cysts and adult flukes. Adult flukes recovered from the pleural cavity or from within cysts (Fig. 1a) were washed three times for 5 min in warm sterile PBS and maintained in warm PBS until they emptied their gut contents. Parasites were incubated for 24–72 h at 37 °C, 5% CO_2_ in 500µL of sterile culture medium (RPMI 1640 supplemented with 30 mM HEPES pH 7.2, 2% glucose and 10% penicillin/streptomycin/amphotericin mix; Sigma, St. Louis MO, USA). Every 12 h, the supernatant containing the ESP was collected under a laminar flow hood and replaced with fresh serum-free medium. The fluid was syringe-filtered through a 0.22 µm filter and stored at − 20 °C until further use. This prevent that voided eggs or worm tissue could contaminate the ESPs. When found, lungs with intact cyst were placed in a sterile petri dish and washed with sterile PBS. After that, an incision in the cyst was performed to retrieve the cyst fluid (CFP) and any parasite inside (Fig. 1a). The inside of the cyst was washed with sterile PBS and all the fluid was collected, syringe-filtered through a 0.22 µm filter and store at − 20 °C until further use.

EV from gerbil blood samples (GBP) were collected from Mongolian gerbils and purified using the ME™ kit for exosome isolation (Vivitide, Gardner, MA, USA). This affinity purification kit binds EV with a peptide (Vn96) that binds to canonical heat shock proteins (HSPs) present in EV the membranes. This binding leads to the EV being easily precipitated into a pellet with a brief series of spins with a benchtop centrifuge following the manufacture instructions.

ESP and CFP EV were purified by size exclusion chromatography (SEC) using IZON qEV columns (iZON Science Ltd, Oxford, UK). The ESP and CFP samples were thawed, pooled and concentrated using an Amicon® Ultra-4 Centrifugal Filter Units, 3 kDa (Merck Millipore, Billerica, MA, US) by spinning at 3000×*g* until the sample volume was 500 µL. The qEV SEC columns were washed in accordance with the manufacturer’s protocol with 10 ml of sterile filtered PBS. This was completed twice to ensure no sodium azide remnant was left in the column. Following this, the sample was loaded onto the loading frit. The buffer volume was collected as the sample moved through the column. Once the sample had fully entered the column (not visible on the loading frit), sterile filtered PBS was added in 0.5 mL increments. Each addition of PBS was collected in a separate “fraction”. Based on work from the manufacturer, it could be predicted that the EV would elute in fractions 5–9. Fractions were pooled and concentrated to 100 µl by use of Amicon filters mentioned above by centrifugation at 3000 × g for 16 min.

### Electron microscopy

*P. kellicotti* ESP EV were fixed with 1% glutaraldehyde (Ted Pella Inc., Redding, CA, USA), and allowed to absorb onto freshly glow discharged formvar/carbon-coated copper grids (200 mesh, Ted Pella Inc., Redding, CA, USA) for 10 min. Grids were then washed two times in dH_2_O, and stained with 1% aqueous uranyl acetate (Ted Pella Inc.) for 1 min. Excess liquid was gently wicked off and grids were allowed to air dry. Samples were viewed on a JEOL 1200EX transmission electron microscope (Jeol, Peabody, MA, USA) equipped with an AMT 8 megapixel digital camera (Advanced Microscopy Techniques, Woburn, MA, USA). EV sizes were then analyzed with ImageJ software^44^, and graphic design was performed using GraphPad Prism, version 6.07 (GraphPad Software Inc., La Jolla, CA, USA).

### LC–MS/MS, sample preparation and peptide identification

Peptides were prepared as previously described using a modification of the filter-aided sample preparation method^45^. Data were acquired from technical and biological replicates for ESP, CFP and GBP sample preparations (Table 4) using a QExactive ™ mass spectrometer (Thermo Fisher Scientific, Waltham, MA, USA) interfaced with an EASY Spray Ion source (Thermo Fisher Scientific) and analyzed as previously described^46^.

**Table 4:** Sample guide.

Sample type	Biological replicates	Technical replicates	Requirements
EV GBP	2	3	Protein detected in ≥ 2 technical or biological replicates with at least 2 peptides
EV CFP	4	2–3	Protein detected in ≥ 2 biological replicates with at least 2 peptides
EV ESP	1	3	Protein detected in ≥ 2 technical replicates with at least 2 peptides

Number of replicates analyzed by proteomics and requirements to call a protein as present. Extracellular vesicles (EV) samples from gerbil blood proteins (GBP), cyst fluid proteins (CFP) and excretion/secretion products (ESP) from *P. kellicotti.*

### Protein functional annotation

A BLASTP search against a *M. unguiculatus* database was performed in order to discard any peptides which exactly matched host peptide sequences (considering leucine/isoleucine to be equivalent). The processed spectra count and peptide count results are available for each condition and sample in Supplementary Tables S1 and S2. Functional annotations for all *P. kellicotti* proteins were assigned using results from InterProScan v5.59-91.0^47^ to identify gene ontology^48^ classifications and InterPro functional domains^49^, and GhostKOALA v2.2^50^ to assign KEGG^51^ annotations. Potentially secreted proteins were identified using SignalP v6.0^52^ for signal peptides and transmembrane domains, where any proteins with a predicted signal peptide and fewer than 2 transmembrane domains was classified as secreted. Additional protein naming was performed using PANNZER^53^ and Sma3s^54^. Functional enrichment was performed using the website http://webgestalt.org/ for KEGG (Kyoto Encyclopedia of Genes and Genomes) and InterPro domains while GOSTATS v2.50 was used for GO (Gene ontology) “molecular function” child term enrichment. Enrichment was considered significant if the FDR-corrected P values were ≤ 0.05, and at least 3 genes/proteins were represented in the enriched group.

### Immunohistochemistry

Adult *P. kellicotti* (day 22 post infection) were fixed in Bouin’s solution, and lung tissue from infected and non-infected gerbils were fixed in 10% Formaldehyde. The samples were embedded in paraffin, sectioned at 5 μm and used as template to localize the cysteine protease-6 (CP6, GENBANK AZZ10060.1) within the fluke (Fig. 2) as well as in tissue of the lung cyst that contains the fluke (Fig. 3). To localize Cp-6 in the parasite tissue a polyclonal mouse antibody raised against recombinant CP-6 was used as primary antibody in a 1:100 dilution using the alkaline phosphatase anti-alkaline phosphatase (APAAP) method described previously^35^. To localize Cp-6 in the host tissue we used the indirect horseradish peroxidase method (HRP) and immunofluorescence to avoid background of endogenous alkaline phosphatase in the lung cyst tissue. For the HRP method, endogenous peroxidase was blocked by incubation with 3% (v/w) peroxidase for 5 min at room temperature. After incubation with the first antibody (Cp-6 1:100), a goat anti-mouse IgG human ads-HRP (Southern Biotech, Birmingham, AL, USA) was used as a secondary antibody. EnVison FLEX HRP Magenta Substrate chromogen System (Dako Omnis, Agilent Technologies, Glostrup Denmark) was used according to the manufacturer’s recommendation. The slides were counterstained with Mayer’s Hematoxylin.

For immunofluorescence, the sections were incubated with Alexa488 (Invitrogen, Carlsbad, CA, USA) as secondary antibody for one hour at room temperature and Wheat Germ Agglutinin (WGA) 633 was used to label cell membranes ProLong Gold antifade reagent with DAPI (Invitrogen) was used as mounting medium.

### Supplementary Information

Supplementary Information 1.

Supplementary Information 2.

## Data Availability

The mass spectrometry proteomics data have been made publicly available through the ProteomeXchange Consortium (http://proteomecentral.proteomexchange.org) via the MassIVE partner repository (reference number PXD041776).
