# Supplementary material for: The proteome of extracellular vesicles of the lung fluke Paragonimus kellicotti produced in vitro and in the lung cyst
Source: Sci Rep. 2023 Aug 22;13:13726. doi: 10.1038/s41598-023-39966-x (PMC10444896; doi:10.1038/s41598-023-39966-x)
Supplement: Supplementary file 1 — Supplementary Information 1. [file 41598_2023_39966_MOESM1_ESM.docx]

Supplementary Figure S1. **Alignment for cysteine protease from *P. kellicotti*.** Sequences accession numbers AZZ10060.1, KAF6777412.1 and KAF6769383.1. In grey are the conserved amino acids within the three sequences.


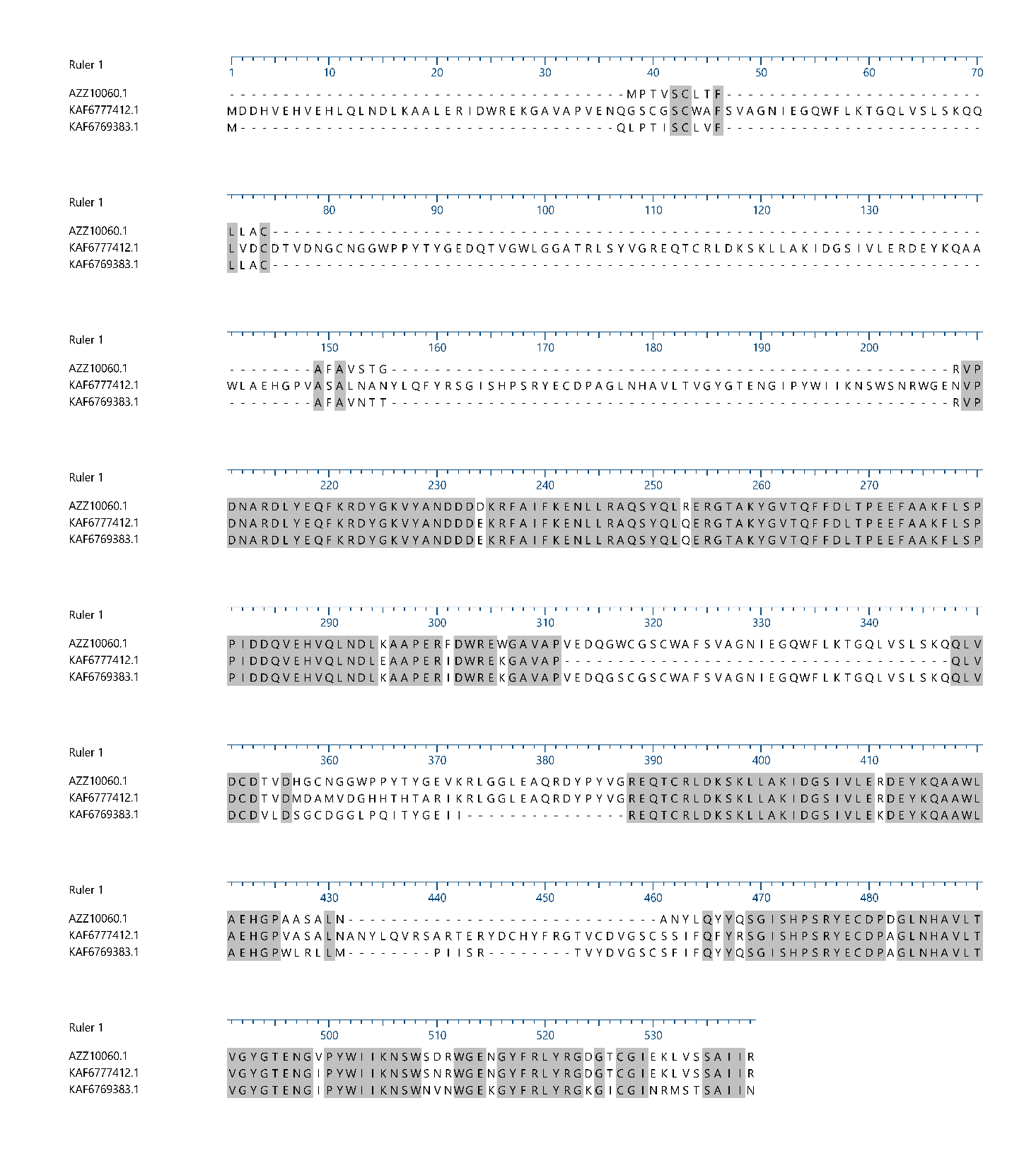


Supplementary Figure S2. ***P. kellicotti*** c**ysteine proteases sequences**. A. Sequence for KAF6777412.1. In red: peptides found with LC-MS/MS. In blue: repeated fragments in the sequence. B. cysteine protease-6 (accession number AZZ10060.1) sequence with the peptides assigned to KAF6777412.1 in the CFP analysis in red. The number show their order in the KAF6777412.1 sequence.

A.


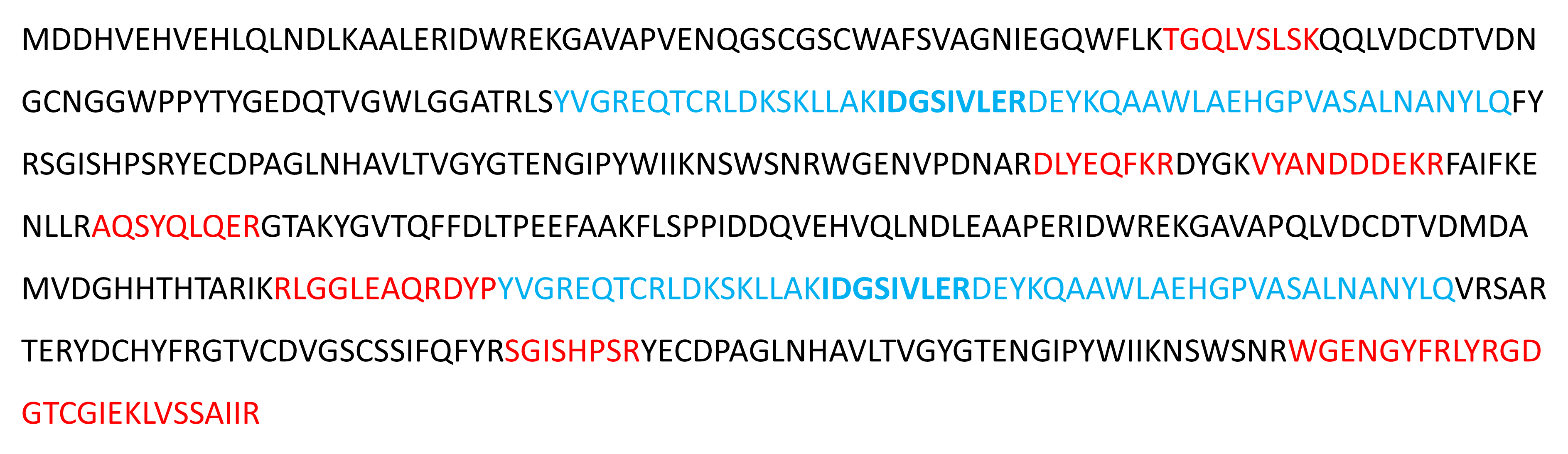


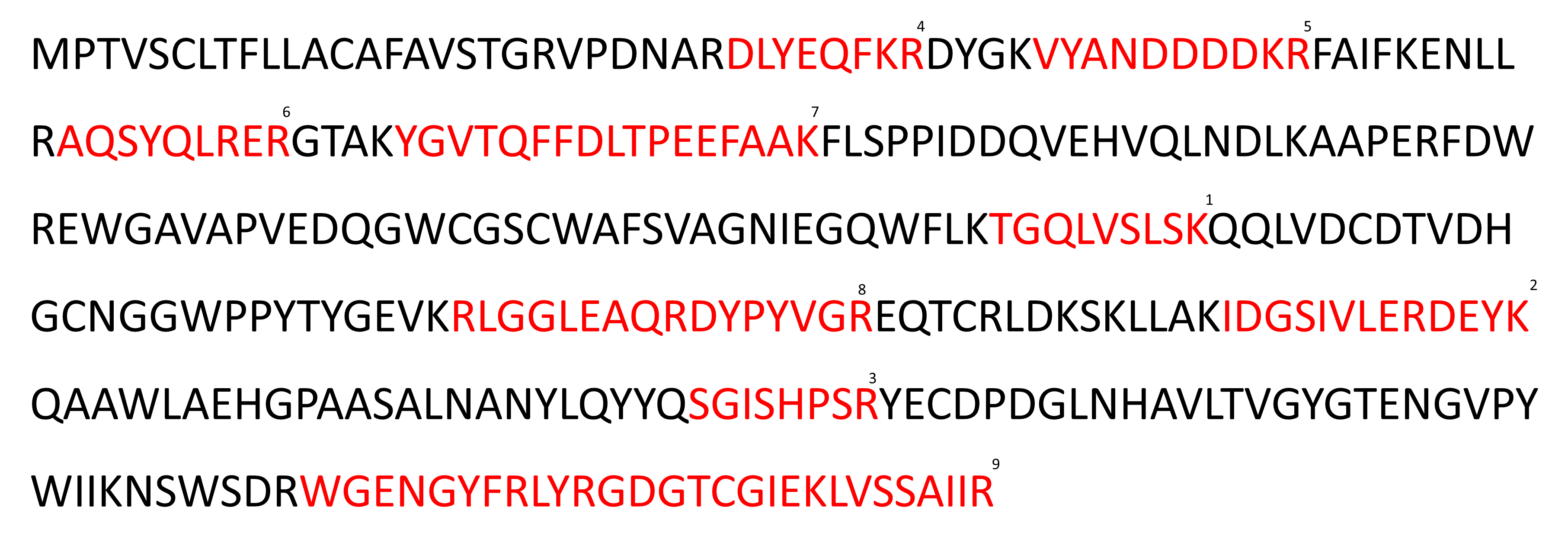
B
